# Transient Ischaemic Attacks in a Girl with Subclavian Steal Syndrome

**DOI:** 10.34763/jmotherandchild.20252901.d-25-00007

**Published:** 2025-08-12

**Authors:** Agnieszka Szmigielska, Michał Buczyński, Aleksandra Śledziewska, Małgorzata-Pańczyk-Tomaszewska, Mariusz Ireneusz Furmanek

**Affiliations:** Department of Pediatrics and Nephrology, Medical University of Warsaw, Poland; Department of Cardiothoracic and Transplantology, Medical University of Warsaw, Poland; Department of Pediatric Radiology, Medical University of Warsaw, Poland

**Keywords:** Transient ischemic attacks, subclavian steal syndrome, hypertension, right-sided aortic arch, vascular anomalies, children

## Abstract

A Transient Ischemic Attack (TIA) in children results from a temporary interruption of blood flow to the brain, leading to brief neurological symptoms. The most common causes of pediatric TIA include congenital heart defects and vascular anomalies. We present a 10-year-old girl with neurological symptoms due to subclavian steal syndrome. Physical examination revealed an asymmetry in blood pressure measurements between the upper limbs, exceeding 30 mmHg. Echocardiography revealed a right-sided aortic arch (RAA) with an atypical configuration of the cephalic vessels. Ultrasound of the vertebral arteries demonstrated reversed flow direction in the left vertebral artery. CT angiography confirmed RAA and an atypical branching pattern. The left subclavian artery was narrower with critical stenosis in its proximal segment, adjacent to the origin of the ductus arteriosus. The girl was qualified to surgical intervention to correct the incomplete vascular ring associated with a RAA and an aberrant left subclavian artery.

## Introduction

A Paediatric Transient Ischaemic Attack (TIA) results from a temporary interruption of blood flow to the brain, leading to brief neurological symptoms. These symptoms typically last from a few minutes to several hours and resolve completely within 24 hours without causing permanent neurological damage [1]. Common clinical manifestations include sudden weakness affecting the face, arm, or leg; dysarthria; aphasia; ataxia; blurred vision; diplopia; transient monocular blindness; vertigo; severe sudden headache; altered mental status; brief memory loss; and transient episodes of unresponsiveness [2]. TIA is a significant warning sign of a potential stroke, necessitating immediate medical evaluation with imaging studies such as Magnetic Resonance Imaging (MRI) and Magnetic Resonance Angiography (MRA) to determine the underlying aetiology and implement preventive measures. The most common causes of paediatric TIA include congenital heart defects, vascular anomalies, haematological disorders, infections, neurological conditions, as well as metabolic and genetic diseases [3].

Subclavian Steal Syndrome (SSS) occurs due to stenosis or occlusion of the subclavian artery, resulting in a reversal of blood flow in the vertebral artery. The condition is more prevalent in older adults, particularly those with atherosclerosis, which is the primary cause of subclavian artery stenosis [4]. Asymptomatic cases of SSS are relatively common, with a prevalence of 1–2% in the general population, while symptomatic cases are less frequent, occurring in 0.6% to 6.4% of individuals undergoing vascular evaluation for cerebrovascular symptoms [5, 6]. Clinical presentation includes dizziness, vertigo, arm pain, and weakness, particularly during upper extremity exertion [7]. SSS is exceedingly rare in children but has been reported in association with congenital heart defects, vascular anomalies, Takayasu arteritis, coarctation of the aorta, and iatrogenic causes such as post-surgical complications [8]. Management of SSS depends on the severity of symptoms and the underlying pathology, with treatment options ranging from medical management and risk factor control to endovascular intervention, including Percutaneous Transluminal Angioplasty (PTA) with stenting, and surgical revascularization in cases of severe or recurrent disease [9].

## Case report

**Figure 1.**
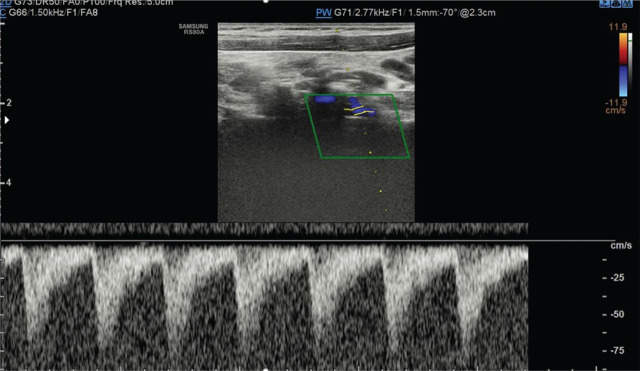
Doppler ultrasound. Measuring gate in the left vertebral artery: reversed flow direction with preserved spectrum.

**Figure 2.**
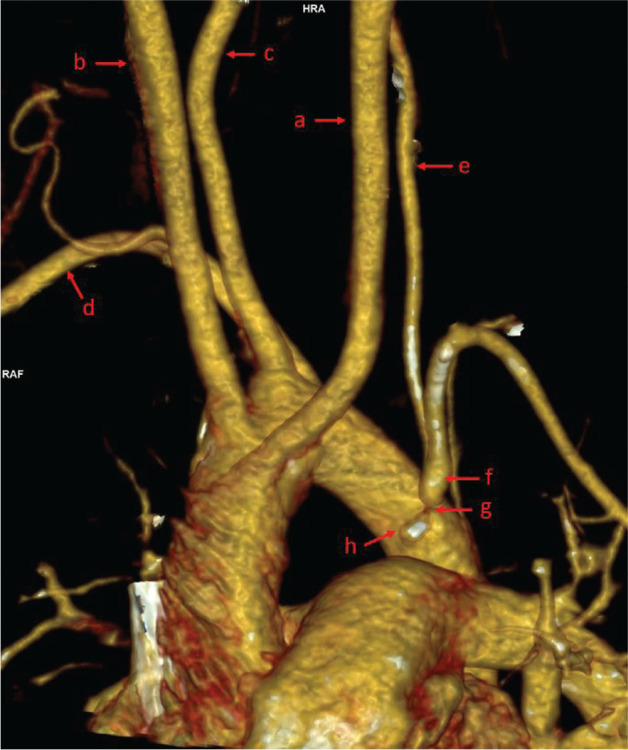
Angio-CT of the cephalad arteries in the spatial projection presentation (VRT). The left common carotid artery (a), the right common carotid artery (b), the right vertebral artery (c), and the right subclavian artery (d) branch off from the aortic arch. The left subclavian artery (f), critically narrowed at the ostium (g), leaves in the immediate vicinity of the origin of the ductus arteriosus (h). The left vertebral body (e) is at least twice as narrow as the contralateral vertebral body (c).

A 10-year-old girl was admitted to the hospital due to elevated blood pressure, reaching 140/80 mmHg, accompanied by neurological symptoms. She was born via caesarean section at 36 weeks of gestation due to placenta previa, following the mother’s seventh pregnancy and sixth delivery, with a birth weight of 2360 g. On the first day of life, she underwent surgery for congenital small intestine obstruction, after which poor weight gain was observed. An upper gastrointestinal X-ray revealed a vascular ring compressing the oesophagus posteriorly at the Th4-5 level. Echocardiography findings were normal. Following dietary modifications, her weight and height gain normalized. She has a history of behavioural disorders and has been monitored in a Psychological Clinic for ADHD and Asperger’s syndrome. Additionally, she has been diagnosed with bronchial asthma due to periodic shortness of breath and is treated intermittently with Montelukast and Salbutamol. Over the past three months, she has reported recurrent headaches, dizziness, transient visual disturbances including periodic blindness in the left eye, and episodic numbness in her hands. Upon admission, her general condition was stable. Physical examination revealed an asymmetry in blood pressure measurements between the upper limbs, exceeding 30 mmHg, a visual defect consistent with myopia, and abdominal scars from the previous gastrointestinal surgery. Routine blood and urine tests were within normal limits. Infections with Lyme disease, Toxoplasma, Toxocara, Epstein-Barr virus (EBV), and Mycoplasma pneumoniae were ruled out. Serum ANA and ANCA antibodies were negative. A lumbar puncture was performed, and cerebrospinal fluid analysis was normal. A 24-hour blood pressure measurement did not reveal hypertension. Laboratory tests showed normal concentrations of metanephrines and thyroid hormones. Imaging studies, including abdominal ultrasound, renal vascular Doppler, thyroid ultrasound, and head CT, were unremarkable. Ophthalmological evaluation did not show any significant abnormalities. Electroencephalography examination revealed a baseline activity of 10–11 Hz waves with amplitudes up to 75 μV. In the posterotemporo-occipital leads, single sharp waves with amplitudes up to 120 μV were observed bilaterally. Fs-leading rhythms began at a frequency of 1 Hz. Hyperventilation induced sharpened slow waves and exacerbated acute changes in the posterior leads, which reverted to the baseline state upon cessation. MRI of the head demonstrated asymmetry of the vertebral arteries, with either agenesis or significant hypoplasia of the left vertebral artery, indicating a vascular anomaly. Given the previously diagnosed vascular ring, neurological symptoms, and blood pressure asymmetry in the upper limbs, steal syndrome was suspected. Echocardiography revealed a right-sided aortic arch with an atypical configuration of the cephalic vessels. Ultrasound of the vertebral arteries demonstrated reversed flow direction in the left vertebral artery while maintaining a typical flow spectrum (Fig. 1). The morphological appearance of the common carotid arteries, internal carotid arteries, and vertebral arteries in the precranial sections was normal, though asymmetry was noted. The right vertebral artery measured up to 5 mm in width, while the left was up to 3 mm. Blood flow in all vessels, except for the left vertebral artery, was directed cephalad with a normal flow spectrum. CT angiography confirmed a right-sided aortic arch and an atypical branching pattern. The left common carotid artery, right common carotid artery, right vertebral artery, and right subclavian artery originated sequentially, followed by the left subclavian artery, which gave rise early to the left vertebral artery. The right subclavian artery measured approximately 6.9 mm at its origin, while the left subclavian artery was narrower with critical stenosis in its proximal segment, adjacent to the origin of the ductus arteriosus. The vertebral arteries exhibited asymmetry, with the right measuring approximately 6.8 mm at its origin and the left approximately 3 mm (Fig. 2). The trachea was compressed at the point where it intersected with the aortic arch. The descending aorta was positioned to the left of the spine.

**Figure 3.**
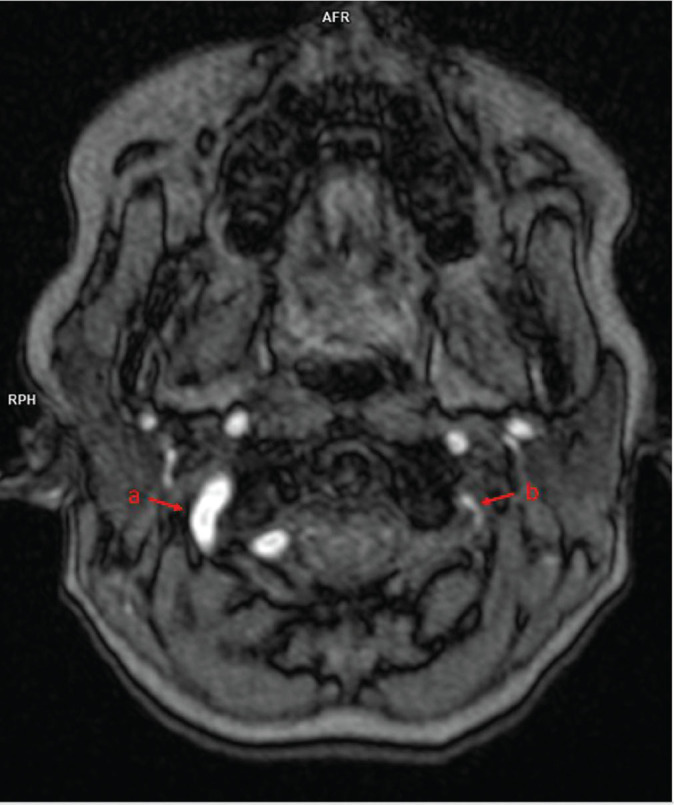
MR angiography of cephalad arteries using the TOF technique. Source image from the level of the C1 vertebra. The right vertebral column (a) is much wider than the left one (b). A high signal indicates the cephalad direction of flow in the vessels.

The patient was deemed a candidate for surgical intervention to correct the incomplete vascular ring associated with a right-sided aortic arch and an aberrant left subclavian artery. During cardiac surgery, the patent ductus arteriosus was ligated, and the ostium of the left subclavian artery was reconstructed via a left-sided thoracotomy. Postoperative follow-up with MRI, including TOF angiography, confirmed restoration of normal cephalic blood flow in the left vertebral artery, with inflow originating from the subclavian artery (Fig. 3). The patient remains under ongoing cardiological and neurological supervision.

## Discussion

Headaches are one of the most common symptoms in children and are becoming more common among school children. The prevalence of headaches increases with age. In 7-year-olds, it is 37–51%, increasing to 57–82% in 15-year-olds [10, 11]. The causes of headache in children can be multifactorial, including stress, dehydration, visual disturbances, and infections. Head imaging is particularly recommended when the headache is severe with sudden onset, persistent, or progressively worsening; associated with head trauma; accompanied by vomiting, confusion, visual disturbances; or when it occurs during sleep and awakens the child.

The presented case of a 10-year-old girl with neurological symptoms highlights the challenges in diagnosing and determining the aetiology of TIA in paediatric patients. Despite an extensive differential diagnosis, no definitive cause was initially identified. Routine laboratory tests and evaluations for systemic and infectious diseases yielded normal results. The neurological symptoms coincided with episodes of hypertension, although ambulatory blood pressure monitoring (ABPM) did not confirm persistent hypertension. Upon re-evaluation of the patient’s history, clinical findings, and disease progression, subclavian steal syndrome (SSS) was suspected. A vascular ring had been previously noted in infancy during an upper gastrointestinal X-ray. The neurological symptoms were characterized by sudden onset during physical exertion and spontaneous resolution within minutes. A significant asymmetry in blood pressure measurements between the upper limbs was observed on physical examination. Doppler ultrasound demonstrated reversed blood flow in the left vertebral artery, while echocardiography revealed a right-sided aortic arch (RAA).

RAA is a rare congenital vascular anomaly in which the aortic arch develops on the right side rather than the left, often resulting in an abnormal branching pattern that may cause vascular compression or subclavian artery stenosis, potentially leading to SSS [12]. Patients with RAA and SSS may experience symptoms related to decreased cerebral perfusion, including dizziness, vertigo, arm pain, fatigue, and, in severe cases, syncope [13]. Blood pressure measurements typically reveal asymmetry, with lower pressure in the affected limb. In severe cases, neurological symptoms may resemble those of TIA or stroke. Doppler ultrasound is a key diagnostic tool in detecting retrograde flow in the vertebral artery, a hallmark of SSS [14]. Symptomatic cases of RAA associated with a vascular ring require surgical correction, including division of the vascular ring, such as the ligamentum arteriosum, to restore normal blood flow [15].

In the described case, surgical intervention was successfully performed to correct the vascular anomaly. Postoperative Doppler ultrasound of the vertebral arteries and MRI of the head confirmed the restoration of normal cephalad blood flow. The patient remains under ongoing observation and has reported no recurrence of neurological symptoms.

## Conclusions

Transient ischaemic attacks in children are rare and often mimic other conditions, making a thorough diagnostic evaluation essential for accurate diagnosis and appropriate management. Subclavian steal syndrome is an uncommon condition in paediatric patients and is typically associated with congenital vascular anomalies. The presence of a right-sided aortic arch increases the risk of developing subclavian steal syndrome due to its potential impact on vascular flow dynamics. In severe cases, surgical intervention is necessary to correct the underlying vascular anomaly and restore normal blood circulation.
